# Comparison of the SuperARMS and ARMS for detecting *EGFR* mutations in liquid-based cytology specimens from NSCLC patients

**DOI:** 10.1186/s13000-019-0910-5

**Published:** 2020-01-31

**Authors:** Wei Wu, Ziyang Cao, Wei Zhang, Liping Zhang, Likun Hou, Chunyan Wu

**Affiliations:** Department of Pathology, Shanghai Pulmonary Hospital, Tongji University School of Medicine, 200433 Shanghai, People’s Republic of China

**Keywords:** *EGFR*, SuperARMS, ARMS, NSCLC, Liquid-based cytology

## Abstract

**Background:**

Non-surgical cytological specimens are adequate not only for accurate histological subtyping but also for molecular profiling. A modified amplification refractory mutation system polymerase chain reaction (ARMS PCR), known as SuperARMS PCR, was improved by optimizing the primers designation, which provides a higher sensitivity and specificity approach for free plasma DNA detection. It is unclear whether SuperARMS PCR detects epidermal growth factor receptor (*EGFR*) mutations in cytology samples. The aim of this study was to compare the EGFR mutations detected by ARMS PCR and SuperARMS PCR in cytology samples derived from advanced non-small cell lung cancer (NSCLC) patients.

**Methods:**

From March 2016 to March 2018, a total of 234 cytological samples were obtained from primary or metastatic lesions of NSCLC, including 144 fine-needle aspirations (FNAs), 36 endobroncheal ultrasonography (EBUS) FNAs, 36 transbronchial needle aspirations (TBNAs) and 18 pleural effusion (PLEs). *EGFR* mutations were simultaneously detected using an ADx-ARMS EGFR kit (Amoy Diagnostics CO., ltd., Xiamen, China) and an ADx-SuperARMS EGFR kit (Amoy Diagnostics CO., ltd., Xiamen, China). Digital droplet PCR (ddPCR) and next-generation sequencing (NGS) were further used to verify the *EGFR* mutant inconsistent samples.

**Results:**

All of the 234 patients with advanced or recurrent NSCLC were diagnosed and assessed by two cytopathologists, and their *EGFR* mutation statuses were successfully detected by ARMS and SuperARMS. Importantly, the SuperARMS and ARMS methods showed a highly concordant result of 94.0% (220/234) (95%CI: 85.0, 95.0%). The positive rate of the SuperARMS was higher than the ARMS in the cytology samples for *EGFR* detection (46.2% vs. 40.2%). The specific *EGFR* mutation sites in 16 samples (6.8%) were not completely consistent between the SuperARMS and ARMS. A total of 14 patients showed *EGFR* mutations when detected by SuperARMS, but by ARMS there were *EGFR* wild-type. Two patients were detected as having one more *EGFR* mutation site by SuperARMS than by ARMS. ddPCR and NGS were used to further confirm the *EGFR* mutations in these inconsistent samples. Eight samples had the same mutation results as the SuperARMS, and 6 samples were not verified because the remaining DNA was insufficient. A total of 78 *EGFR* mutation patients received Tyrosine Kinase Inhibitor (TKI) treatment. The overall objective response rate (ORR) was 88.5% (69/78) for *EGFR* TKI treatment.

**Conclusion:**

SuperARMS showed a high sensitivity and specificity for *EGFR* detection and thus, is expected to become a routine test in the clinic to be used as a widely available, easy-to-operate and sensitive method for *EGFR* mutation detection in liquid-based cytology samples.

## Introduction

Lung cancer remains the most common cause of cancer death worldwide. Approximately 70% of patients with non-small cell lung cancer (NSCLC) come to clinical attention at an advanced stage [1]. Small biopsy or cytological specimens may be a good choice in these patients, while they have no surgical options. The types of non-surgical cytological samples include fine-needle aspiration (FNA), endobronchial ultrasound-guided (EBUS) biopsy, transbronchial needle aspiration (TBNA), bronchoscopic brush (BB) and pleural effusion (PLE), which are reliable for the diagnosis and staging of thoracic malignancy, especially of NSCLC [2–6].

Many investigators report that cytological material obtained with minimally invasive procedures is adequate not only for accurate histological subtyping but also for molecular profiling [7–9]. In fact, studies have demonstrated optimal results using a multitude of cytological samples for molecular tests [10–12]. In our previous study, epidermal growth factor receptor (*EGFR*) mutation detection was performed by ARMS in liquid-based cytology samples from patients with NSCLC and their paired tissue samples, and the results of the two groups were identical [13]. Cytology specimens provide high quality DNA for the evaluation of clinically relevant mutations. Research groups, such as Allegrini S and Malapelle U, have also directly used liquid-based cytology samples for *EGFR* and Kirsten rat sarcoma viral oncogene (*KRAS*) mutation detection with better results [14, 15]. Under an era of individualized targeted therapy for NSCLC, it is crucial, for guiding treatment decisions, to obtain informative cytological material for both diagnosis and molecular testing. In our lab, we have increasingly used cytology specimens for molecular testing when cytology material was the only specimen source available.

Amplification Refractory Mutation System (ARMS) is an improved polymerase chain reaction (PCR) system and is an important platform for the detection of genes that drive NSCLC. The AmoyDx *EGFR* 29 Mutations Detection Kit is approved by the China Food and Drug Administration (CFDA) for the clinical testing of *EGFR* mutations using tissue samples. Recently, a novel technique called SuperARMS, which is a modified version of ARMS that optimizes the primers designation, was shown to provide a high sensitivity and specificity approach for free plasma DNA detection. No studies have been performed to compare ARMS and SuperARMS for *EGFR* mutation using cytology specimens. Therefore, it is unclear whether SuperARMS can be used to detect *EGFR* mutations in cytology samples and if it improves the sensitivity compared with ARMS. Thus, we conducted the present study to compare the *EGFR* mutations detected by ARMS and SuperARMS PCR in cytology samples derived from advanced NSCLC patients.

## Materials and methods

### Cytology specimens and study design

From March 2016 to March 2018, a total of 234 patients with advanced NSCLC were retrospectively enrolled in this study at the Shanghai Pulmonary Hospital, Tongji University. All the cytological samples were obtained from primary or metastatic lesions of NSCLC and included 144 FNAs, 36 EBUS FNAs, 36 TBNAs and 18 PLEs. The imaging data were independently reviewed by the authors to evaluate their treatment responses according to the Response Evaluation Criteria in Solid Tumors (RECIST) version 1.1. Progression-free survival (PFS) was calculated from the date of the initiating tyrosine kinase inhibitors (TKI) treatment to a radiologic or clinical observation of the disease progression.

**Fig. 1:**
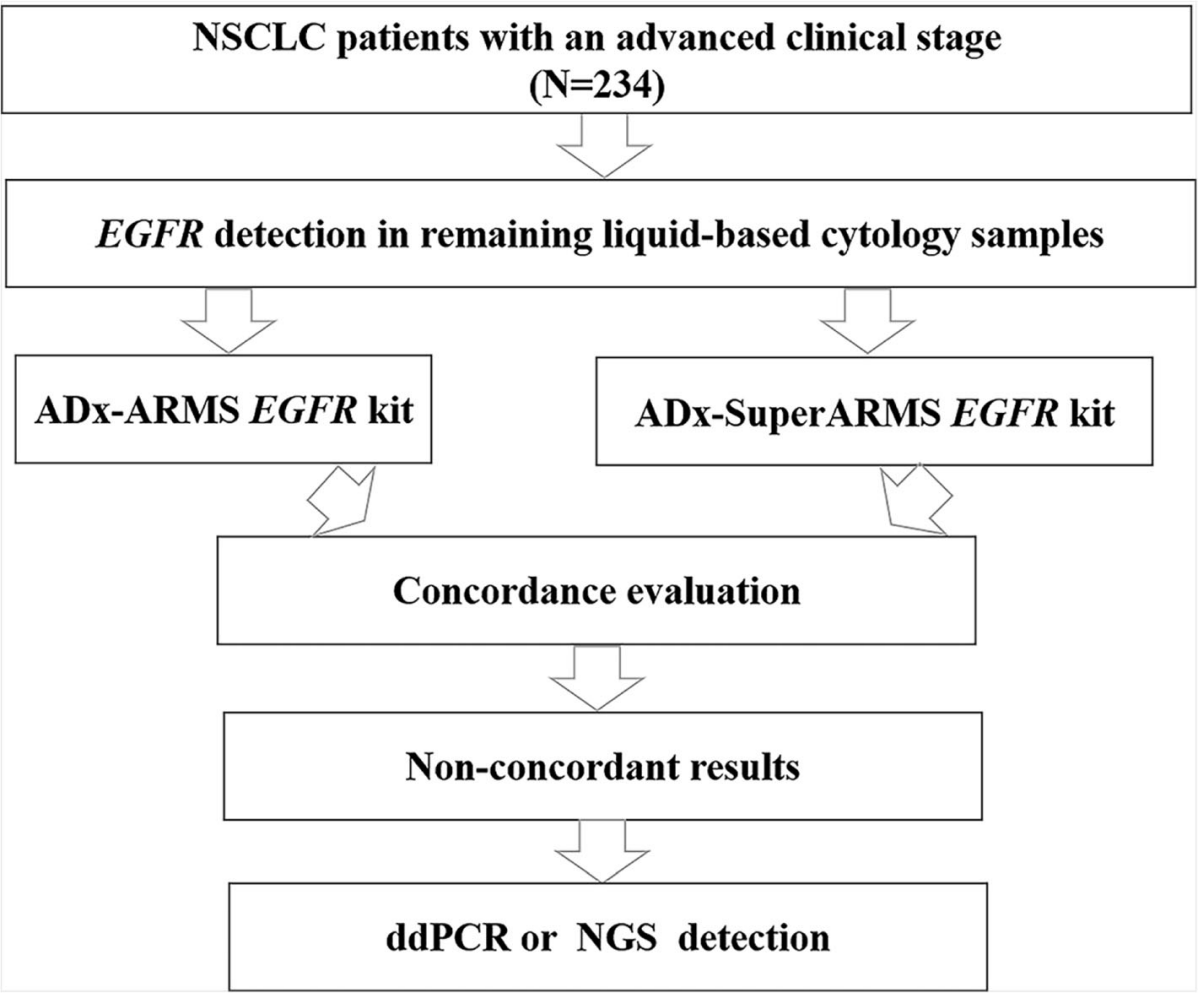
A flowchart describing the study design Abbreviations: NSCLC = non-small cell lung cancer; *EGFR* = epidermal growth factor receptor; NGS = next-generation sequencing.

A flowchart describing the study design is presented in Fig. 1. The study protocol was approved by the Institute Review Board of the Shanghai Pulmonary Hospital.

### Sample collection

The liquid-based cytology specimen preparations were handled according to the standard specimen processing protocols in our laboratory. Cytology samples from FNAs, EBUS FNAs, TBNAs and PLEs were taken by clinicians and sent to pathology department for Thinprep cytologic test (TCT) within 1 day. One ThinPrep slide was stained with hematoxylin and eosin (H&E) from the various cell types and was reviewed for morphologic evaluation by two cytopathologists. The remainder of the specimen liquid-based cytology specimens were rinsed in CytoLyt solution (Hologic) and were centrifuged to generate a cell pellet stored at 4 °C. If the H&E–stained smear was positive for NSCLC, we quantitated the number of tumor cells as more than 200 cells by the H&E–stained smear and observed the residual cell pellet, which was visible to the naked eye, to ensure that the specimen was adequate for *EGFR* molecular testing.

### DNA extraction

The residual cell pellets were used for the DNA extraction, which was performed using a Tissue DNA Kit (Amoy Diagnostics Co., Xiamen, China), following the manufacturer’s instructions. The optical density of the extracted DNA samples was measured using a microplate spectrophotometer (Biotek). The A260/A280 value of all the samples was 1.8 to 2.1. The extracted DNA was used for EGFR molecular testing.

### *EGFR* mutation detection

The *EGFR* mutations in the residual liquid-based cytology samples were simultaneously detected using an ADx-ARMS *EGFR* kitand an ADx-SuperARMS *EGFR* kit, according to the manufacturer’s instructions. Briefly, the DNA templates were added to the *EGFR* Reaction Mix, which included the primers, probes, dNTPs, buffer, Mg2+ and Taq DNA polymerases. The PCR reactions were performed on a Stratagene Mx3000P quantitative PCR (qPCR) system. After the PCR reaction was completed, the data interpretation was conducted according to results interpretation criteria of the *EGFR* mutation detection kit.

### ddPCR detection

The ddPCR was performed according to the manufacturer’s instructions. Briefly, the mutant reaction solution was prepared, and then, the emulsified microdroplets were generated in the QX200TM Droplet Generator instrument and were put into a 96-well plate for amplification. The PCR reaction conditions were as follows: incubation at 95 °C for 10 min, followed by 45 cycles of 95 °C for 15 s and 60°Cfor 60 s. After the PCR amplification, the 96-well plate was placed in the QX200 microdrop analyzer, and the data analyses were conducted using Quanta Soft analysis software.

### NGS detection

DNA sequencing was carried out using a capture-based sequencing panel (Amoy Diagnostics CO., Ltd., Xiamen, China). The kit covered the targeted drug-related hot spot mutation regions of 10 genes, including *EGFR* et al. The liquid-based cytology DNA samples were sequenced with a NextSeq 500 (Illumina, Inc), with pair-end reads, with a reading length of PE150 and a sequencing depth of > 10,000x. The sequence data were analyzed using the AmoyDx NGS Data Analysis System ADXLC10 module (Amoy Diagnostics CO., Ltd., Xiamen, China).

### Statistical analysis

The statistical analyses were performed with SPSS, version 17.0 (IBM, Armonk, NY). A two-sided tailed Fisher’s exact test was applied to the data for one subgroup with a count less than 5. *p* < 0.05 was considered significantly different.

## Results

### Patient characteristics

A total of 234 patients with advanced or recurrent NSCLC were retrospectively enrolled into the study. The patients’ clinical characteristics are listed in Table 1. The median age was 65 years (range, 31–85 years). The patients consisted of 79 women and 155 men. Eighty-four patients were smokers, 145 were never-smokers, and 5 were unclear smokers. Fifty-eight patients were classified as at stage III, and 176 were at stage IV. The liquid-based cytology sample types included: FNA from the lungs for 114 patients, FNA from the lymph nodes for 30 patients, EBUS for 36 patients, TBNA for 36 patients and PLE for 15 patients. All the specimens were confirmed by two cytopathologists, including 172 adenocarcinoma, 21 squamous and 41 NSCLC.

**Table 1:** Clinical characteristics (*n* = 234)

Factors	No.(%)
Sex	
Male	155(48.7%)
Female	79(48.7%)
Age, years	
Median	65
Range	31–85
Smoking history	
Never	145(62.0%)
Former/current	84(35.9%)
Unclear	5(2.1%)
Pathology	
Adenocarcinoma	172(73.5%)
Squamous	21(9.0%)
NSCLC	41(17.5%)
Tumor Stage	
III	58(24.8%)
IV	176(75.2%)
Cytological sample types	
FNA (lung)	114(48.7%)
FNA (lymph node)	30(12.8%)
EBUS FNA	36(15.4%)
TBNA	36(15.4%)
Pleural effusion	18(7.7%)

### Comparisons of the SuperARMS and the ADx-ARMS for detecting *EGFR* in the cytological specimens

All 234 liquid-based cytology samples were successfully detected for their *EGFR* mutation status by ARMS and SuperARMS. The results are shown in Table 2. When tested by ARMS, 94 of 234 (40.2%) patients were identified to have an *EGFR* mutation. When using the SuperARMS, 108 of 234 (46.2%) patients were demonstrated to have an *EGFR* mutation. The positive rate of the SuperARMS was higher than the ARMS in the cytology samples for *EGFR* detection (46.2% vs. 40.2%). The two-sided tailed Fisher’s exact test showed that this was a significant difference (*p* < 0.05). The negative results and the positive results from the SuperARMS were in accordance with the ARMS results, at 90.0% (95% CI, 91.0,97.1%) and 100.0% (94/94), respectively. The concordance rate of the *EGFR* mutation status between SuperARMS and ARMS was 94.0% (220/234) (95%CI:85.0,95.0%). Fourteen patients showed an *EGFR* mutation by SuperARMS but were *EGFR* wild-type by ARMS. In addition, two patients were detected with one more *EGFR* mutation site by SuperARMS than by ARMS.

**Table 2:** Comparison of *EGFR* mutation status using ARMS and SuperARMS (N = 234)

		ARMS		Total
		Mt	Wt	
SuperARMS	Mt	94	14	108
	Wt	0	126	126
	Total	94	140	234
	PCR*	100% (94/94)		
	NCR*	90%(126/140)(95%CI:91.0,97.1%)		
	Overall agreement*	94%(220/234)(95%CI:85.0,95.0%)		

*percentage [95% confidence interval (CI)];

Mt: mutation; Wt: wild type; PCR: positive coincident rate; NPV: negative coincident rate

### SuperARMS for detecting specific *EGFR* mutation types

Out of total of 234 samples, 16 cases were discordant between SuperARMS and ARMS (Table 3). Among them, 14 (87.5%) cases were detected as positive for *EGFR* mutations only by SuperARMS, while the other two samples showed different mutations by these two methods. We performed ddPCR and NGS to further confirmed the inconsistent samples(*n* = 12/16, 4 samples were insufficient). 1 sample with *EGFR* 20-ins mutation (Sample ID:FK020) is beyond capability of ddPCR, so 11/12 samples were identified by ddPCR at first. Then we successful detected 5 sample using NGS (Table 3). 2 samples were negative for mutations。 2 samples had a special L858R mutation type (NM_005228.3: exon21:c.2573 T > G:p.L858R), which was only detected by NGS and SuperARMS. This observation indicates that SuperARMS and NGS methods can detect more sites of mutation than ARMS in the tested regions.

**Table 3:** Sixteen Nonconcordant Cases of SuperARMS and ARMS for Cytology *EGFR* Detection

Sample ID		ARMS	Super-ARMS	ddPCR	NGS
FK008	19-DEL	19-DEL/T790 M	19-DEL/T790 M	NA	
FK020	WT	20-INS	NA	20-INS	
FK055	WT	L858R	WT	NE DNA	
FK062	WT	L858R	WT	NM_005228.3:exon21:c.2573_2574delinsGT:p.L858R	
FK066	WT	L858R	WT	NM_005228.3:exon21:c.2573 T > G:p.L858R	
FK080	WT	19-DEL	19-DEL	NA	
FK108	WT	L858R	L858R	NA	
FK129	WT	L858R	NE DNA	NE DNA	
FK130	WT	L858R	NE DNA	NE DNA	
FK135	WT	19-DEL	19-DEL	NA	
FK147	WT	19-DEL	WT	WT	
FK166	WT	L858R	WT	NE DNA	
FK190	WT	19-DEL	WT	WT	
FK206	19-DEL	19-DEL/L858R	NE DNA	NE DNA	
FK220	WT	20-ins	NE DNA	NE DNA	
FK221	WT	19-DEL/L858R	19-DEL/L858R	NA	

*NE DNA: not enough DNA

### *EGFR* mutation status and prediction of *EGFR*-TKI efficacy

**Fig. 2:**
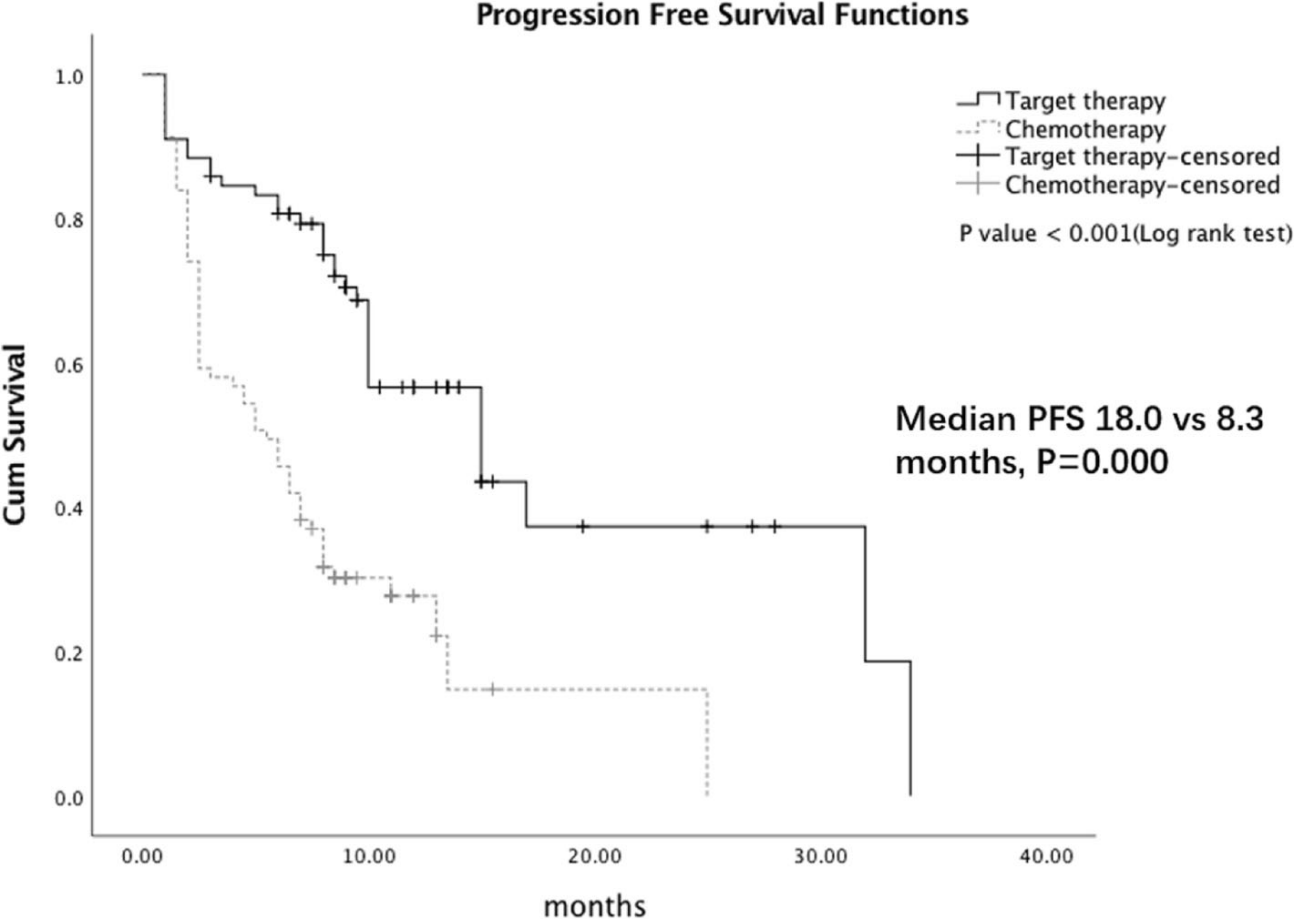
PFS in *EGFR* mutation patients compared with *EGFR* wild-type patients

As shown in Table 4, out of the 94 positive *EGFR* mutation patients by ARMS, 77 patients received *EGFR*-TKI treatment. In another 14 patients with an *EGFR* mutation by SuperARMS, only 1 patient received *EGFR*-TKI treatment. The ORR was 88.5% (69/78) for *EGFR* TKI treatment. In patients with or without *EGFR* mutation, 81 received chemotherapy. The median PFS was significantly prolonged in the *EGFR* mutation patients compared with the *EGFR* wild-type patients (18.0 months vs 8.3 months, *P* < 0.01) (Fig. 2). These data suggested that *EGFR* mutation status in cytology samples detected by ARMS and SuperARMS is predictive of tumor response to *EGFR*-TKIs and survival results.

**Table 4:** *EGFR* Mutation Status and TKIs treatment

	Sample No.	Positive No.	Negtive No.	Sensitive MT No.	targeted therapy No.
ARMS	234	94	140	92	77
SuperARMS	234	108	126	104	78

## Discussion

Currently, the molecular spectrum of tumor patients promotes the development of individualized treatment. Especially in patients with NSCLC, the mutation status of *EGFR* has great guiding significance for the use of TKIs [16]. Now more than ever, obtaining adequate material for the molecular testing of NSCLC is crucial. In the clinical setting, a cytological specimen may be the only material available for the determination of *EGFR* mutation status. Liquid-based cytology specimens have been used for molecular assays with optimal results, although rare large series have been published.

It has been reported that the number of tumor cells in the remaining liquid base cell sample varies significantly between the different samples, with limited sensitivity of the ARMS detection in low tumor cell content samples [17]. SuperARMS is a highly sensitive kit that detects 41 *EGFR* mutations in exons 18 to 21 in human plasma cfDNA samples. To the best of our knowledge, ours is the first study to evaluate the performance of SuperARMS in liquid-based cytology samples for *EGFR* detection.

The feasibility of conventional smears and the remaining liquid-based cytology samples for molecular detection has been evaluated in multiple laboratories [18–20]. Gilda da Cunha Santos’s team compared the nucleic acid quality of the remaining liquid-based cytology samples, smears and cell blocks for molecular detection, and found that well-preserved liquid-based cytology samples had a good nucleic acid yield and quality [21]. Maurizio Martini’s research team explained the role of cytological samples in tumor diagnosis, which is not only used in the diagnosis, but also in molecular testing, and a small amount of high-quality nucleic acid provides effective molecular genetic features [22]. Consistent with these reports in the literature, our liquid-based cytology samples had good DNA quality and or the *EGFR* gene status was successfully detected using SuperARMS and ARMS.

In this study, various cytological specimens, including FNA, EBUS FNA, TBNA and PLE, were all subjected to the *EGFR* mutational analysis using SuperARMS and ARMS, and 100% (234/234) of the samples were suitable for testing. Firstly, the SuperARMS and ARMS methods showed highly concordant results, at 94.0% (220/234) (95%CI: 85.0, 95.0%). Moreover, SuperARMS detected more mutations compared with ARMS. Our study showed that SuperARMS, as a highly sensitive molecular detection method, can be used for the detection of *EGFR* not only in plasma ctDNA, but also in cytological samples DNA.

The high sensitivity and specificity of SuperARMS has been demonstrated in a series of liquid biopsy studies [23, 24]. Our study confirmed that SuperARMS had high sensitivity and specificity in liquid-based cytology samples. The positive rate of SuperARMS was higher than ARMS in the cytology samples for EGFR detection (46.2% vs. 40.2%) (Table 2). Our study showed that SuperARMS was not only used in the blood, but also in liquid-based cytology samples. Is there a false positive result in the SuperARMS? We analyzed the reasons for the inconsistency of the results of the validation samples. The false positive results caused by the contamination of aerosol in the laboratory should be excluded. Notably that two samples had the special L858R mutation type (NM_005228.3: exon21:c.2573 T > G:p.L858R), which was detected by SuperARMS and NGS. The specific reason for this observation is that the SuperARMS and NGS methods detect more sites.

Many previous studies report that there is a significant correlation between *EGFR* mutation status and treatment for PFS [25, 26]. Consistent with these studies, our study revealed that patients with *EGFR* mutations, using targeted drugs as the first line of treatment, had a longer PFS compared to patients without *EGFR* mutations, using chemotherapy drugs as the first line of treatment, as detected by both ARMS and SuperARMS.

In conclusion, the SuperARMS had a high sensitivity and specificity for *EGFR* detection in liquid-based cytology samples, and these results were good predictors of the efficacy of *EGFR*-TKIs. Thus, the SuperARMS is expected to become a test that is used routinely in the clinic as a widely available, easy-to-operate and sensitive method for *EGFR* mutation detection in liquid-based cytology samples.

## Data Availability

The raw data are available upon request.

## Abbreviations

20-ins: 20 exon insertion mutation
ARMS: amplification Refractory Mutation System
BB: bronchoscopic brush
EBUS: endobronchial ultrasound-guided
EGFR: epidermal growth factor receptor
FNA: fine-needle aspiration
H&E: hematoxylin and eosin
NSCLC: non-small cell lung cancer
ORR: objective response rate
PCR: polymerase chain reaction
PFS: progression-free survival
PLE: pleural effusion
RECIST: response Evaluation Criteria in Solid Tumors
TBNA: transbronchial needle aspiration
TKI: tyrosine kinase inhibitor
